# Domain-mediated interactions for protein subfamily identification

**DOI:** 10.1038/s41598-019-57187-z

**Published:** 2020-01-14

**Authors:** Heetak Lee, Inhae Kim, Seong Kyu Han, Donghyo Kim, Jungho Kong, Sanguk Kim

**Affiliations:** Department of Life Sciences, Pohang University of Science and Technology, Pohang, 790-784 Korea

**Keywords:** Functional clustering, Genome informatics, Protein analysis

## Abstract

Within a protein family, proteins with the same domain often exhibit different cellular functions, despite the shared evolutionary history and molecular function of the domain. We hypothesized that domain-mediated interactions (DMIs) may categorize a protein family into subfamilies because the diversified functions of a single domain often depend on interacting partners of domains. Here we systematically identified DMI subfamilies, in which proteins share domains with DMI partners, as well as with various functional and physical interaction networks in individual species. In humans, DMI subfamily members are associated with similar diseases, including cancers, and are frequently co-associated with the same diseases. DMI information relates to the functional and evolutionary subdivisions of human kinases. In yeast, DMI subfamilies contain proteins with similar phenotypic outcomes from specific chemical treatments. Therefore, the systematic investigation here provides insights into the diverse functions of subfamilies derived from a protein family with a link-centric approach and suggests a useful resource for annotating the functions and phenotypic outcomes of proteins.

## Introduction

Protein families consist of evolutionarily, functionally, and structurally relevant proteins^1^, which provide insights into protein functions and the relationship between genes and phenotypes^2–6^. Protein subfamilies emerged to divide protein families into subgroups of diversified molecular functions; hence, each subfamily includes functionally more similar proteins. Many previous studies, including subfamily classifications in phylogenomics (SCI-PHY)^7^, Genome Modeling and Model Annotation (GeMMA)^8^, Active Sites Modeling and Clustering (ASMC)^9^, Two-Level Iterative Clustering Process (TuLIP)^10^, and Signature Dynamics of Protein Families (SignDy)^11^, utilize the similarity between proteins based on sequence, structure, or dynamics to identify protein subfamilies. As a result of previous classification methods, the functionally relevant proteins within the same subfamily tend to have similar structures and sequences, which may mediate distinct protein interactions across subfamilies within the same protein family^12^.

Here, we propose that particular domain-mediated interactions (DMIs) subdivide a protein family into its subfamilies consisting of biologically relevant proteins. Indeed, functional activities of proteins are closely associated with processes in which interacting proteins are involved^13,14^. For instance, although Pho85 and Bck1 are single-domain proteins and both possess a Pkinase domain (Pfam-A, PF00069), Pho85 interacts with Pcl1 (cyclin) and acts in the cell cycle mechanism, whereas Bck1 interacts with and activates Mkk2 (mitogen-activated kinase kinase) in a cell wall modulation pathway in yeast^15,16^. Similarly, changes in a shared domain across proteins within the same protein family mediate the divergence of interactions^17^. Furthermore, the interacting proteins in a domain-domain interaction are known to co-evolve^18^ and to be functionally similar^19,20^. In previous work, we showed that proteins connected by domain-domain interactions tend to form specific biological modules such as functional groups, protein complexes, and subcellular localization^21^. Thus, protein interactions mediated by the same domain may be an appropriate metric by which to classify subfamilies. However, the implication of using DMIs for dividing protein subfamilies has not yet been systematically investigated.

In this study, we systematically identified DMI subfamilies consisting of proteins sharing the same DMIs in species-specific domain-resolved protein interaction networks (DPNs). We then evaluated the qualification of DMI subfamilies as functional subdivisions of the protein family. We generated 15 independent sets of DMI subfamilies from 15 DPNs based on functional or physical networks in human, yeast, fly, and plant genomes. We found that proteins within the same DMI subfamily tended to be associate with similar functions. Moreover, we compared DMI subfamilies to the functional subfamilies, designated using the kinase classification, and confirmed that members of DMI subfamilies frequently co-occurred in kinase subfamilies. In addition, proteins within the same kinase groups utilized more similar DMIs than did proteins from different kinase groups. In yeast, we sought to identify the relationships between domain interaction interfaces and chemical compounds, and we confirmed these relationships using protein structures. Our results suggest that DMI information contributes to the identification of a group of biologically relevant proteins as a subdivision of protein families and provides insights into a link-centric point of view in inferring the biological roles of proteins.

## Results

### DMI subfamilies are functional subdivisions of their parent-domain families

**Figure 1:**
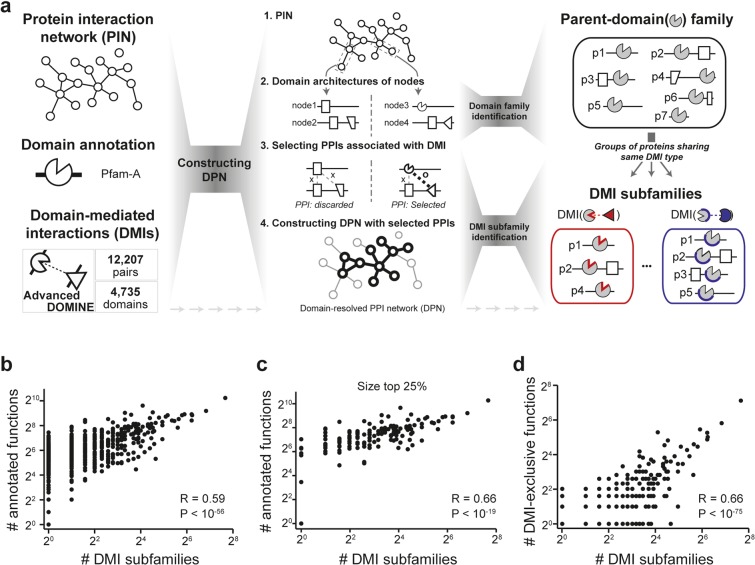
Subcategorization of parent-domain family. (**a**) Scheme for the detection of DMI subfamilies considering domain-mediated interactions from parent-domain family. To construct domain-resolved protein interaction networks (DPNs), species-specific protein interaction networks (e.g., BIOGRID network in humans), domain annotations, and reference domain interactions were used (left). When a protein interaction was connected with at least one DMI, this protein interaction was selected as a link in the DPN (middle). In the genome, proteins sharing a domain were involved in the same parent-domain family (upper right). Among parent-domain family members, proteins sharing a domain interaction were selected as a DMI subfamily (lower right). (**b,c**) Correlation between the number of annotated functions of parent-domain family members without duplicates and the number of DMI subfamilies contained in the parent-domain family. **(b**) Correlation for all parent-domain subfamilies. (**c**) Correlation for the largest 25% of parent-domain families. (**d**) Correlation between the number of DMI-exclusive functions and the number of DMI subfamilies within the parent-domain families.

To investigate the biological relevance among proteins sharing DMIs, we systematically identified DMI subfamilies from domain-resolved protein-protein interaction (PPI) networks. Specifically, we defined a DMI subfamily, ***SF***(*i*, *j*), as a group of proteins that satisfies the following conditions: i) the member proteins share domain *i*; ii) the members have at least one PPI neighbor with domain *j*; iii) domains *i* and *j* interact with each other. We also defined a parent-domain family, ***F***(*i*), as a set of proteins that have domain *i* in a species. Note that ***F***(*i*) is a parent-domain family of ***SF***(*i*, *j*) because ***SF***(*i*, *j*) is always a subset of ***F***(*i*) and ***F***(*i*) needs to contain at least one ***SF***(*i*, *j*). For this identification, we constructed domain-resolved PPI networks (Fig. 1a) in which each PPI connects two proteins with at least one pair of interacting domains. We summarized statistics for such networks in Supplementary Table S1. We used 12,207 pairs of interacting domains that had been identified by either 3D structures of protein complexes or various prediction methods^21,22^. We applied this approach to 15 PPI networks and generated 15 sets of DMI subfamilies (see **Methods**). For example, from the human-BIOGRID network, we detected 3,620 DMI subfamilies from 630 parent-domain families (Supplementary Table S2).

We found that DMI subfamilies are functional subgroups of their parent-domain families. We observed that the number of DMI subfamilies significantly correlated with the number of functional annotations within the parent-domain families (Fig. 1b; Spearman correlation coefficient, R = 0.59, *P* < 10^−56^). We also confirmed this result based on functional annotations excluding gene-function associations bearing the IPI evidence code (inferred from physical interactions) (see **Methods**) (Fig. S1a; R = 0.58, *P* < 10^−55^). These results indicate that parent-domain families with greater functional diversity tend to be divided into more DMI subfamilies. We observed such a positive correlation (Fig. 1c; R = 0.66, *P* < 10^−19^) for the largest 25% of parent-domain families, which contained more than 27 members (Fig. S2a); the results were consistent in other quarters (Fig. S2b–d; *P* < 0.05).

We also found that DMI subfamilies characterized protein functions that were unexplained by their parent-domain families. We defined such functions as DMI-exclusive functions and summarized them in Supplementary Table S3. The existence of DMI-exclusive functions indicates that DMI information clusters the dispersed parent-domain family proteins that perform in the same biological process or pathway. The number of DMI subfamilies was significantly correlated with the number of DMI-exclusive functions (Fig. 1d; R = 0.66, *P* < 10^−75^). For example, exocytosis (GO:0006887) is a DMI-exclusive function in ***F***(Ras), which is enriched with ***SF***(Ras, FYVE_2) consisting of RAB8B, RAB27A, RAB27B, RAB8A, HRAS, NRAS, RAB11A, RAB3A, and KRAS. These Rab GTPases regulate steps of vesicle transports. In particular, RAB27A interacts with two FYVE_2 domain-containing proteins, SYTL1-5 and RPH3A, which are effectors that function in the dynamics of secretory granules^23^. On the other hand, ***SF***(Ras, RhoGEF) consists of 15 proteins, including 10 Rho GTPases. This DMI subfamily is enriched with a DMI-exclusive function, regulation of cell migration (GO:0030334). We also found that the number of DMI subfamilies correlated with the number of DMI-exclusive functions based on functional annotation excluding gene-function associations bearing the IPI evidence code (Fig. S1b; R = 0.61, *P* < 10^−63^). Furthermore, we found that the number of associated DMIs was positively correlated with the functional diversity of genes (Fig. S3; R = 0.23, *P* < 10^−67^). Taken together, these results suggest that DMI information reduces the functional heterogeneity of parent-domain families.

### DMI subfamilies contain functionally and phenotypically relevant proteins

**Figure 2:**
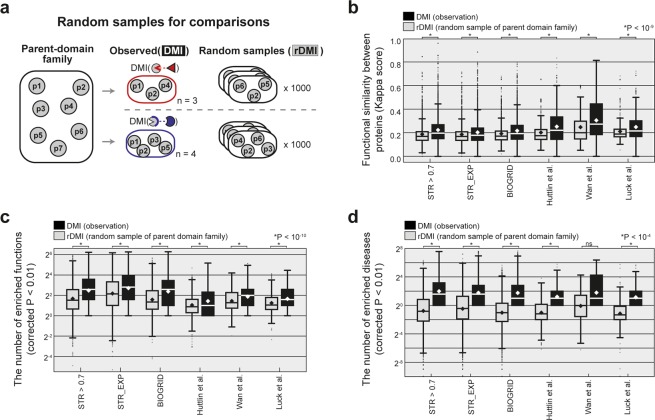
DMI subfamilies as biological subcategories of parent-domain families. (**a**) Scheme for preparation of comparisons between subcategories of a parent-domain family: rDMI is a random sample of a parent-domain family with size N, which is the number of members of the real DMI subfamily. For each DMI subfamily, we sampled 1,000 times from a parent-domain family to prepare an rDMI set for comparison. (**b–d**) Comparisons between DMI subfamily and random samples of parent-domain family. Gray and black boxplots indicate distributions of values from random samples and DMI subfamily, respectively. Diamonds show mean; *p*-values were calculated by Wilcoxon signed-rank test. Several networks were used to detect DMI subfamilies: STR > 0.7 **(a** STRING network containing links with a combined score greater than 0.7); STR_EXP (**a** STRING network containing links with an experimental score greater than zero); BIOGRID (involving experimentally-confirmed links); and Huttlin *et al*., Wan *et al*., and Luck *et al*. (networks constructed through AP-MS, co-fractionation, and yeast two-hybrid, respectively). (**b**) Functional similarity between members within each subfamily (*P < 10^−9^). (**c**) The number of enriched functional terms (*P < 10^−10^). (**d**) The number of enriched diseases (*P < 10^−4^).

We next investigated the biological relevance of DMI subfamily members using two types of functional similarity measurements: the kappa score and the number of enriched terms (see **Methods**). The kappa score (κ) represents the functional similarity between members and quantifies the extent to which two genes share categorical items, considering the agreement expected by chance^24^. The number of enriched terms (*N*_*E*_) indicates how many functions are commonly associated with the members of a DMI subfamily. A subfamily with higher κ and *N*_*E*_ values contains functionally more relevant proteins than a subfamily with lower κ and *N*_*E*_ values. Furthermore, we compared the values from DMI subfamilies with the values from random DMI (rDMI) subfamilies. For example, for a subfamily, ***SF***(*i*, *j*), which contained four members, we randomly sampled four proteins from the parent-domain family, ***F***(*i*), for 1,000 times (Fig. 2a). In humans, DMI subfamily members consistently had higher κ scores than the members of rDMI subfamilies across six comparisons (Fig. 2b; Wilcoxon signed-rank test, *P* < 10^−9^). For example, in the co-fractionation network (‘Wan *et al*.’ on the figure), average κ scores of DMI and rDMI were 0.305 and 0.247, respectively (*P* = 5.29 × 10^−10^). We also confirmed the same tendency in sets of DMI subfamilies from yeast, fly, and plant genomes using the species-specific networks STR > 0.7, STR_EXP, and BIOGRID, respectively (see **Methods**) (Fig. S4; *P* < 10^−9^).

Furthermore, when we investigated the number of enriched functions (*N*_*E*_) in DMI subfamilies and compared this with the *N*_*E*_ in rDMI subfamilies, we recognized that DMI subfamilies had a higher *N*_*E*_ in all of the sets from the given networks (Fig. 2c; *P* < 10^−10^). We also found the same tendency in other sets of DMI subfamilies from the other nine networks (Fig. S5; *P* < 10^−25^). In addition, because we were concerned about the potential bias raised by GO annotations such as “inferred from physical interaction” (IPI), we tested the number of enriched functions using the functional annotations except gene-function annotations bearing the IPI code and confirmed the same tendency (Fig. S6; *P* < 10^−6^). To confirm the robustness of our findings, we investigated *N*_*E*_ based on gene-disease associations (**see Methods**). We found that DMI subfamilies were enriched with more diseases than rDMI subfamilies in all comparisons except the co-fractionation network (Fig. 2d; Supplementary Table S4; *P* < 10^−4^). Moreover, DMI subfamilies within the same parent-domain family were distinctly related to cancer types (Supplementary Table S5, Hypergeometric test, Corrected *P* < 0.01). For example, ***SF***(Pkinase, tRNA-synt_1c), ***SF***(Pkinase, I-set), ***SF***(Pkinase, FGF), ***SF***(Pkinase, DSPc), and ***SF***(Pkinase, Death) associated with melanoma, breast carcinoma, skin neoplasms, squamous cell carcinoma, and lung neoplasms, respectively. For instance, ***SF***(Pkinase, Death) contains seven members (MAP3K8, MAPK3, MAPK14, MAPK1, AKT1, CHEK2, and DAPK1) that are related to lung neoplasms. Indeed, Fas-associated protein with death domain (FADD) is upregulated in lung adenocarcinomas, and expression of phosphorylated FADD correlated with poor clinical outcomes^25^. This indicates that FADD needs to interact with kinases, and kinases may collaborate with particular substrates in distinct cancer types. Taken together, the results suggest that DMI subfamilies can be regarded as functional and phenotypic subcategories of parent-domain families.

Because the identity of shared PPI partners is a powerful aid in detecting groups of biologically relevant proteins, we next investigated how many DMI subfamilies were associated with multiple-shared PPI partners. Conceptually, DMI subfamily members may interact with multiple proteins through a corresponding DMI (Fig. S7a). For example, ***SF***(DEAD, PROCT) contains seven proteins including DExH-Box Helicases (DHXs), and these proteins commonly interact with PRPF8, which possesses a PROCT domain. We found that in human datasets, 6.6% to 10.8% of DMI subfamilies have a single PPI partner from the given networks (Supplementary Table S6). For instance, from the BIOGRID network, 6.6% (238 out of 3,620) of DMI subfamilies have a single PPI partner. This indicates that most DMI subfamilies (89.2% to 93.4%) have multiple-shared PPI partners, and DMI information provides a distinct boundary for the identification of protein groups. Furthermore, we investigated κ and *N*_*E*_ for sets of DMI subfamilies classified by the number of shared PPI partners (n): DMI-A (n = 1) and DMI-B (n > 1). Almost all comparisons showed that DMI subfamilies have significantly higher κ than rDMI subfamilies (*P* < 0.001) apart from the AP-MS network (‘Huttlin *et al*.’ on the figure) DMI-A case (Fig. S7b; *P* = 0.002). DMI subfamilies also have significantly higher *N*_*E*_ (function) than rDMI subfamilies in all the given comparisons (Fig. S7c; *P* < 0.001). Although on average DMI subfamilies have higher *N*_*E*_ (disease) than rDMI subfamilies, DMI-A shows significance only in the AP-MS network. However, DMI-B shows significance in almost all networks except for the co-fractionation network (Fig. S7d; *P* < 0.001), and these results show the same tendency as the disease results presented in Fig. 2d.

### DMI information is relevant to the functional classification of kinases

**Figure 3:**
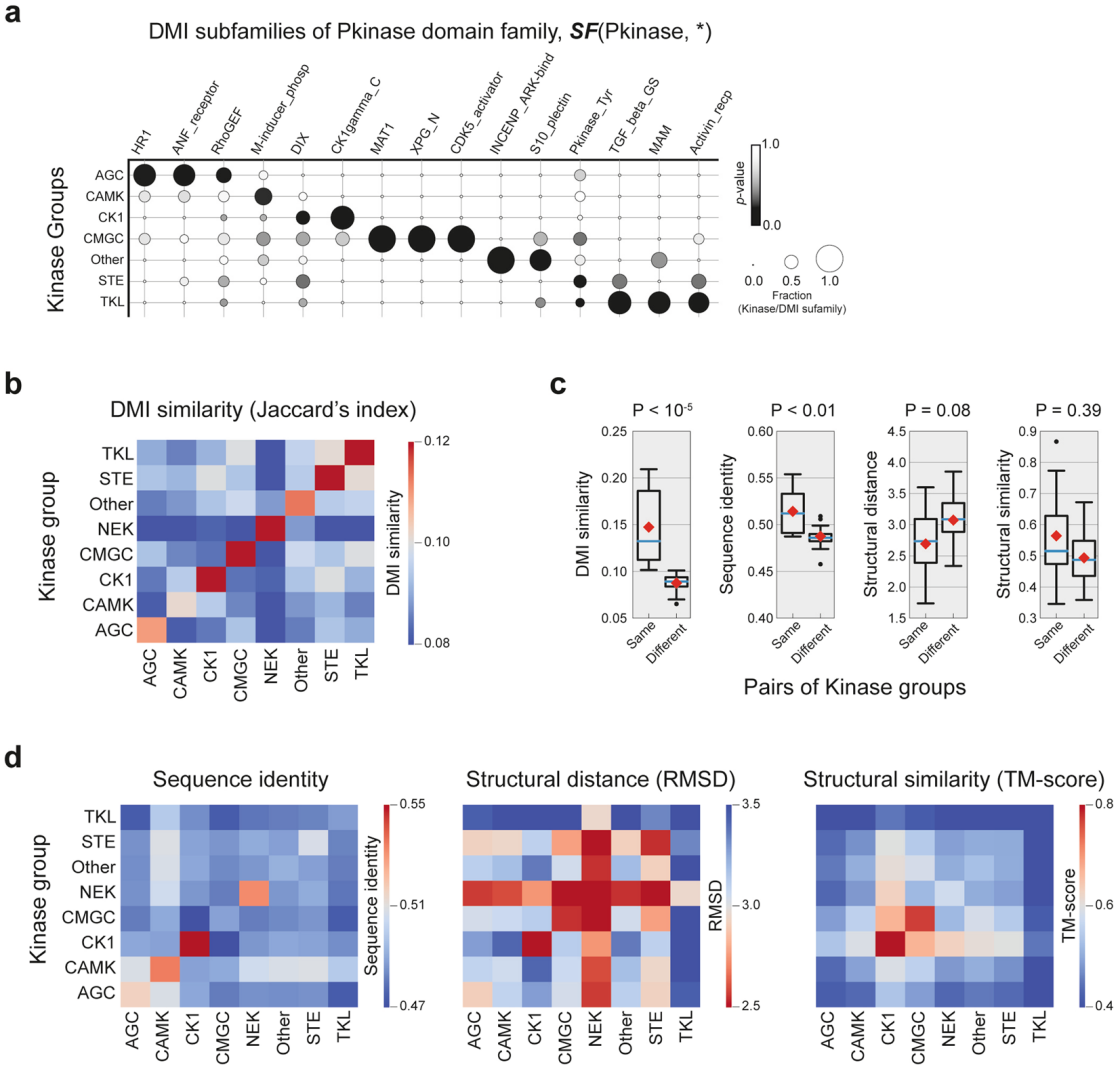
Relationship between kinase groups and DMI subfamilies. (**a)** Overlap analysis between the kinase group and child DMI subfamilies of Pkinase (PF00069) domain family. DMI subfamilies, enriched with at least one kinase group (*P* < 0.01 and top three DMI subfamilies), were represented on a matrix. Circle size shows the overlap ratio between kinase group and DMI subfamily. Gradient indicates the empirical *p*-value of the overlap ratio. (**b–d**) Similarity matrices between kinase groups. The similarities are calculated distinctly according to the features of members such as DMI profile similarity, sequence identity, structural distance, and structural similarity. Each cell of a matrix contains the average similarity between members within the kinase group (diagonal terms) or across kinase groups (off-diagonal terms). (**b**) The similarity between kinase group members based on their associated DMIs. The color bar shows similarities (red: similar; blue: dissimilar). (**c**) Comparison of the average similarity between distributions from diagonal terms and off-diagonal terms. Higher values indicate higher similarity for DMI similarity, sequence identity, and structural similarity. Lower values indicate higher similarity for structural distance. The *p*-values were calculated by the Mann-Whitney U test. Diamonds indicate mean; blue lines show median of distributions. (**d**) Similarity matrices based on sequence identity (left), structural distance (middle), and structural similarity (right).

To test our hypothesis with well-known functional subfamilies, we investigated overlaps between DMI subfamilies of ***F***(Pkinase) from the BIOGRID network and kinase groups in human kinase classification. Manning *et al*. (2002) classified kinases into subfamilies using substrate specificity and functional similarity of kinases^26,27^. The kinase groups are functionally divergent subfamilies of kinases, and each kinase group contains biologically relevant kinases. We found that ***SF***(Pkinase, *) members significantly co-occurred in individual kinase groups, including the protein kinase A, G, and C group (AGC), the glycogen synthase kinase and CDK-like kinase group (CMGC), the calcium- and calmodulin-regulated kinase group (CAMK), and a group of sterile kinases (STE) (Fig. 3a; empirical *P* < 0.01). The overlap ratio is defined as the number of overlapping kinases between a DMI subfamily and a kinase group divided by the number of members in a given DMI subfamily (see **Methods**). For example, ***SF***(Pkinase, MAT1), consisting of CDK2, CDK7, CDK8, and ICK, is significantly enriched with only the CMGC kinase group. All ***SF***(Pkinase, MAT1) members interact with MNAT1, which possesses a targeting factor for CDK-activating kinase, the MAT1 domain^28^. In another example, ***SF***(Pkinase, TGF_beta_GS) is significantly enriched with the tyrosine kinase-like (TKL) group. Among members of this DMI subfamily, BMPR2 phosphorylates the glycine/serine rich domain (TGF_beta_GS domain, PF08515) contained in BMPR1A and BMPR1B^29^. These results and examples indicate that there is a substantial agreement between kinase classification and DMI subfamilies of ***F***(Pkinase).

We detected DMI subfamilies using individual DMIs that proteins use to form protein interactions. However, to compare DMI information with sequence and structure features, we calculated similarities between proteins using DMI profiles as DMI similarity. Each DMI profile consists of the Pkinase domain (PF00069)-mediated interactions associated with a protein. We found that kinases within the same kinase group tended to be close to each other in dendrograms based on DMI similarity (Fig. S8). We also estimated average DMI similarities of kinase pairs within and across kinase groups (see **Methods**). The DMI similarities of kinase pairs within kinase groups (diagonal terms) were significantly higher than those of kinase pairs across kinase groups (off-diagonal terms) (Fig. 3b,c; *P* < 10^−5^). DMI similarity was greater for proteins within the same kinase subfamilies than for proteins between different subfamilies, and the difference was more distinguishable compared to sequence identity (*P* < 0.01), structural distance (RMSD, *P* = 0.08), and structural similarity (TM-score, *P* = 0.39) (Fig. 3c,d). Note that higher DMI similarity, higher sequence identity, higher structural similarity (TM-score), and shorter structural distance (RMSD) indicate that the compared proteins are biologically more similar. Taken together, these results suggest that the functional subcategorization of kinases is related to DMI information rather than to the structure of the kinases.

### DMI subfamilies contribute to the identification of the interaction between DMI interface and chemical compound

**Figure 4:**
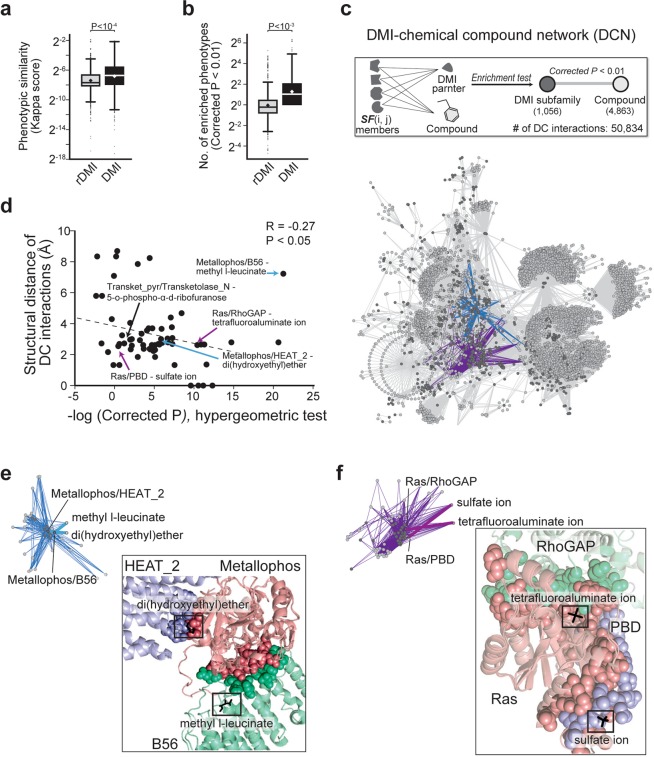
Associations between DMI subfamily and chemical compounds. (**a,b**) Phenotypic relevance of members within the same DMI subfamily in yeast. Diamonds indicate mean; lines on boxplot indicate the median of distributions. The *p*-values were calculated by Wilcoxon signed-rank test. (**a**) Phenotypic similarity between members with kappa scores. (**b**) The number of enriched phenotypes. The relationship between DMI subfamily and phenotype with a corrected *p*-value under 0.01 was selected. A hypergeometric test was used to perform enrichment test; *p*-values were corrected with Bonferroni correction. (**c**) DMI subfamily-chemical compound network. As one application of DMI subfamily, the interaction between domain interaction interface and the chemical compound was suggested by enrichment test between DMI subfamily and compound-protein interaction. Dark gray and gray circles indicate DMI subfamily and chemical compounds, respectively. Link shows the significantly enriched association between DMI subfamily and compound (DC interactions). Blue and purple links show examples. (**d**) Correlation between the structural distances of DC interactions and enrichment significance for DC interactions within reference set. (**e,f**) Examples of DC interactions with three-dimensional structures. Thick links are described by the 3D-structure view. (**e**) The DC interactions mediated by Metallophos domain. (**f**) The DC interactions mediated by Ras domain.

Chemogenomic experiments in yeast have identified genotype-phenotype relationships by measuring fitness defect (FD) score, defined as growth fitness change between a mock-treated deletion strain and a chemical compound (e.g., tunicamycin, 25 μM)-treated deletion strain. Positive or negative FD scores suggest that the deletion strain is sensitive or resistant to the compound, respectively^30^. We determined κ and *N*_*E*_ values for DMI subfamilies using gene-phenotype associations from chemogenomic experiments. We found that DMI subfamilies tended to have higher κ (Fig. 4a; *P* < 10^−4^) and *N*_*E*_ (Fig. 4b; *P* < 10^−3^) values than did rDMI subfamilies. This finding indicates that the DMI subfamily subdivides the parent-domain family based on yeast phenotype.

Likewise, the discovery of physical associations between protein interaction interfaces and chemical compounds is useful information for identifying potential drug targets and for understanding the action of drugs in associated functions and phenotypes^31–33^. We attempted to determine whether a DMI subfamily could assist in identifying the interfaces influenced by chemical compound binding. From simple protein-chemical compound relationships, we tried to infer the associations between domain interaction interfaces and chemical compounds. We constructed a DMI-chemical compound network (DCN) composed of DMI subfamily and compound interactions (DC interactions) defined as enrichment tests relying on protein-compound relationships (Fig. 4c; hypergeometric test, corrected *P* < 0.01). The DC interactions identified the associations between domain interaction interfaces and chemical compounds. The DCN is provided in Supplementary Table S7 (see **Methods**).

As a result, we collected 88 significant adjacent relationships between domain interaction interfaces and chemical compounds within 3D structures as the reference-DC interactions (Fig. S9; structural distance < 10 Å, permutation test *Z* < −1.65). The values and structure information for the reference-DC interactions are summarized in Supplementary Table S8. The statistically more stringent DC interactions showed shorter distances in protein structures (Fig. 4d; R = −0.27, *P* < 0.05). This suggests that the DMI subfamily may be useful in understanding which particular region of a protein mediates the protein-drug interaction and in identifying potential drug targets such as proteins and protein interactions. For example, the Inhibitor_I9/Petidase_S8-tetraethylene glycol interaction had a structural distance (*D*) of 3.99 Å and an enrichment *p*-value (*P*_*E*_) of 0. The Transkey_pyr/Transketolase_N-5-o-phosphono-α-d-ribofuranose interaction had *D* = 2.60 Å and *P*_*E*_ = 0.0002 (Fig. 4d, black arrows, and Fig. S10). Interestingly, we found a domain that possessed multiple interfaces for interacting with different domains and found that these interfaces associated with distinct chemical compounds. The distinct interfaces on Metallophos domain to interact with HEAT_2 domain and B56 domain were close to di(hydroxyethyl)ether (*D* = 3.00 Å, *P*_*E*_ = 1.35 × 10^−6^) and methyl l-leucineate (*D* = 7.23 Å, *P*_*E*_ = 5.79 × 10^−22^), respectively (Fig. 4d, blue arrows, and Fig. 4e). Additionally, the interfaces on Ras domain to interact with RhoGAP domain and PBD domain were close to tetrafluoroaluminate ion (*D* = 2.62 Å, *P*_*E*_ = 3.61 × 10^−11^) and sulfate ion (*D* = 2.66 Å, *P*_*E*_ = 0.27), respectively (Fig. 4d, purple arrows, and Fig. 4f).

## Discussion

We systematically identified DMI subfamilies and confirmed that they subcategorized their parent-domain families in individual species with respect to biological processes and phenotypic outcomes (Figs. 1 and 2). It is plausible that DMI subfamilies are the evolutionary consequence of functional divergence emerged from interaction rewiring. It has previously been suggested that altering partners of a conserved domain would facilitate functional innovation during evolution^17,34^. Indeed, even proteins harboring the same domain, such as kinases, have recruited various other proteins as interacting neighbors and have evolved to carry out distinct molecular functions and biological processes^12^. Furthermore, although DMI subfamilies and evolutionary groups of kinases were consistent within a species, the two sets do not match exactly (Figs. 3 and S7). This indicates that even though the region used for phosphorylating substrates in kinases is highly conserved, kinases utilize distinct DMIs in different species. Taken together, our results suggest that DMI information provides insights into the species-specific diversification of protein families by inferring the interacting partners in a species.

Combinations of domains within a protein have been regarded as the functional building blocks of proteins. Similarly, we have confirmed that domain-mediated interaction information is utilized as a boundary to subdivide a protein family. Conceptually, when a protein has multiple domains (i.e., the domain combination), this protein may belong to several parent-domain families. From each parent-domain family, we identified DMI subfamilies by considering only the interacting domain of the corresponding domain. In other words, DMI subfamilies within different parent-domain families were detected irrespective of the domain architecture of individual proteins. Thus, DMI subfamilies may contain both single-domain and multiple-domain proteins.

Although we focused on the diversified functions of a domain by examining its neighbors, we also observed that proteins were often categorized into multiple DMI subfamilies, which may be indicative of the pleiotropic nature of a protein. Interestingly, recent studies of disease etiology have shown that protein interactions are often more precise functional units for a disease phenotype than a protein itself^35^. For instance, site-directed mutagenesis studies have confirmed that the disruption of a particular interaction can result in a specific phenotype, which is one of many phenotypes associated with a protein^36^. Recently, hypotheses based on this link-centric approach have provided more precise interpretations of genotype-phenotype relationships than gene-centric studies^37–39^. In our dataset, proteins show a positive correlation between the numbers of associated DMIs and the numbers of annotated functions (Fig. S3). This indicates that domain-mediated interactions may be the functional units of proteins. Thus, we expect that our approach will provide insights into expanding protein annotations using a link-centric point of view.

Domain-mediated interactions contain both domain-domain interactions (DDIs) and domain-linear motif interactions (DLIs). In particular, DLIs are crucial for fine regulation of signaling pathways and for connecting two different functional modules^21^. Although DLIs are critical for understanding biological processes, we consider only DDIs because of limitations in the accuracy of DLI datasets and linear motif annotations. Thus, we expect that in the near future, the DMI approach will be applied to the subcategorization of domain families with advanced DLI and linear motif annotations.

Incorporating a large dataset of protein-chemical interactions, we inferred the associations between DMI interfaces and chemical compounds, which were strongly supported by three-dimensional structures (Fig. 4). Notably, the discoveries of druggable PPIs and interfaces are crucial in utilizing non-enzyme proteins involved in signaling pathways, chaperoning, and other critical functions to improve drug responses and reduce adverse effects^40^. Because structural information between PPI interfaces and small molecules is often lacking, various approaches including molecular docking^41^, homology modeling^42^, machine learning^43^, and co-evolution-based statistical modeling^44^ have been used in attempts to infer the druggable interfaces^45^. Our approach provides a large collection of DMI-compound associations (Supplementary Table S7) and relevant biological functions (Supplementary Table S3), which would be useful for the discovery of novel druggable PPIs.

The DMI approach to identifying biological subdivisions of a protein family depends on protein interaction networks and domain interaction networks. Thus we considered two kinds of prediction-based networks such as STRING and BIOGRID and three less biased networks based on experiments such as AP-MS^46^, co-fractionation^47^, and yeast two-hybrid^48^. We have confirmed, in all of the 15 given networks, that DMI information functionally subdivides protein families based on domain annotation. However, the prediction-based networks provide considerably more DMI subfamilies than the less biased networks (Supplementary Table S2). This suggests that the enhancement of networks in coverage and accuracy will promote the novelty of the DMI approach in detecting more and better groups of biologically relevant proteins.

## Methods

### Data collection

#### Protein interaction networks

The STR > 0.7 and STR_EXP networks are functional STRING networks consisting of links with a combined score greater than 0.7 and with an experimental score greater than zero, respectively. All STRING networks were retrieved in August 2019 from the STRING database (https://string-db.org/cgi/download.pl)^49^. The BIOGRID networks consist of links with experimental evidence such as “Affinity Capture-Luminescence”, “Affinity Capture-MS”, “Affinity Capture-RNA”, “Affinity Capture-Western”, “Co-fractionation”, “Co-localization”, “Co-purification”, “FRET”, “PCA”, “Two-hybrid”, “Biochemical Activity”, “Co-crystal Structure”, “Far Western”, “Protein-peptide”, “Protein-RNA”, and “Reconstituted Complex”. We retrieved BIODGRID networks (v3.5.175) in August 2019 from the BIOGRID database (https://downloads.thebiogrid.org/Bio-GRID/Release-Archive/BIOGRID-3.5.175/)^50^. We retrieved human protein interaction networks from the corresponding research based on experiments such as AP-MS^46^, co-fractionation^47^, and yeast two-hybrid^48^.

#### Domain annotations

Domain annotations (Pfam-A^51^) were retrieved in August 2019 from Ensembl Biomart. For human (*H. sapiens*) and fly (*D. melanogaster*) domains, we used “Ensembl Genes 97-GRCh38.p12” and “Ensembl Genes 97-BDGP6.22” to access species-specific annotations, respectively. For yeast domains (*S. cerevisiae*), we used “Ensembl Fungi Genes 44-R64-1-1” in “Ensembl Fungi”. For plant domains (*A. thaliana*), we used “Ensembl Plants Genes 44-TAIR10” in “Ensembl Plants”.

#### Domain-domain interaction

This dataset is based on the DOMINE database (https://manticore.niehs.nih.gov/cgi-bin/Domine). Domain-domain interactions were selected by re-evaluating each predictor in that database^21,52^.

#### Gene ontology

Gene Ontology annotations were retrieved in August 2019 from Gene Ontology Consortium (http://current.geneontology.org/products/pages/downloads.html)^15^. We selected gene-function terms with experimental evidence codes such as “Inferred from Experiment” (EXP) and “Inferred from High Throughput Experiment” (HTP). Independently, we also constructed another set of gene-function annotations excluding the annotation “Inferred from Physical Interaction” (IPI), involved in the “EXP” evidence category, to test whether the IPI code affects the results.

#### KEGG pathways

Protein-KEGG pathway associations were retrieved in August 2019 from Ensembl Biomart^53^. This dataset was also retrieved following the above procedures (i.e. domain annotation).

#### Disease phenotypes

We downloaded the curated gene-disease associations (DisGeNET version 5.0, May 2017) including datasets from UNIPROT, CTD (human subset), PsyGeNET, Orphanet, and HPO from the DisGeNET database (http://www.disgenet.org/downloads)^54^.

#### Kinase classification

We estimated similarities between kinase groups based on DMI profiles, sequence identities, and structural similarity. The kinase classifications were retrieved in August 2019 from the Uniprot database (https://www.uniprot.org/docs/pkinfam.txt)^55^. This classification is based on the human kinome (http://kinase.com)^27^.

#### Human protein sequences

Protein sequences were retrieved in August 2019 from the Uniprot database (http://ftp.ebi.ac.uk/pub/databases/reference_proteomes/QfO/Eukaryota/ UP000005640_9606.fasta.gz)^55^.

#### Human protein structures

To map protein-protein structure associations, we downloaded pdbtosp.txt in August 2019 from the Uniprot database (https://www.uniprot.org/docs/pdbtosp)^55^.

#### Yeast phenotypes

We used datasets from the HaploInsufficiency and Homozygous Profiling (HIPHOP) database(http://chemogenomics.pharmacy.ubc.ca/hiphop/)^56^. This database provides novel correlations between single-gene deletions and chemical environments. Briefly, we mapped gene-condition relationships for relationships having a fitness defect score (FD) >3.5. The FD score reflects the sensitivity of a single-gene deletion strain for a particular chemical compound. Although the direct relationships between genes and chemical environments are not explicit, the phenotypic effects of single-gene deletions and chemicals are correlated. We retrieved 5,888 genes, 3,354 chemicals, and 163,798 gene-to-chemical relationships from the database.

#### STITCH database

From the STITCH database (http://stitch.embl.de/cgi/download.pl)^57^, we downloaded 4932.actions.v5.0.tsv for yeast and identified protein-chemical compound relationships with a combined score greater than 0.7. We mapped chemical compound IDs (single compounds (CIDs) and merged compounds (CIDm)) to SMILES strings (simplified molecular-input line-entry system) using chemicals.v5.0.tsv.

### Subfamily detection

#### Construction of domain-mediated PPI networks

Before detecting DMI subfamilies, we built up the domain-mediated PPI networks using three types of information: domain annotations, reference domain-domain interactions, and protein interaction networks. First, we mapped Pfam-A domains from gene-domain annotations. Second, we prepared the dataset for interacting domain pairs as adopted in our previous work^21^. Finally, from each PPI network, to construct the domain-resolved PPI networks, we selected PPIs connecting two proteins with at least one pair of interacting domains.

#### Detection of parent-domain families

Before applying protein interaction networks, we defined parent-domain families by using domain annotations in each species. This approach has been used to analyze groups of proteins sharing the same domain^58^. Because DMI subfamilies have particular criteria such as a size greater than two, a parent-domain family must contain at least four proteins and one DMI subfamily.

#### Detection of DMI subfamilies

From the domain-mediated PPI network, we detected DMI subfamilies, which consist of proteins sharing the same DMI. Each DMI subfamily contains at least three proteins and includes fewer members than the parent-domain family. However, DMI subfamily members may interact with different proteins, even if those proteins belong to the same DMI subfamily. Thus, we also classified DMI subfamilies with single-shared PPI partners or with multiple-shared PPI partners and investigated the biological relevance of their members.

### Kinase group analysis

To investigate the overlap between kinase groups and DMI subfamilies, we used well-defined kinase classifications. We calculated the overlap ratio by dividing the number of shared members between a DMI subfamily and a kinase group by the total number of members of the DMI subfamily. The *p*-value for the overlap ratio is measured by determining the number of random values that are higher than the observed value, out of 10,000 random values from size-preserved subsets of the parent-domain family.

### Clustering and dendrograms

A dendrogram is a powerful visualization method for trees based on distances between genes or proteins^11,59,60^. Each protein has a particular profile consisting of 0 (not associated with a DMI) or 1 (associated with a DMI). Using a pair of DMI profiles, proteins were clustered by the Ward variance minimization algorithm, which defines the distance between clusters as how much the sum of squares increases when the clusters are merged^61^. We made clusters using the scipy.cluster.hierarchy.linkage function in Python (method = “ward”). Then we converted the hierarchy to Newick tree format and drew a dendrogram using the ETE toolkit^62^ in Python.

### Sequence identity

We calculated sequence identity with the following equation:

1 $$Sequence\,identit{y}_{A,B}=\frac{n(identical\,peptide)}{{\rm{\min }}(len(A),\,len(B))}$$

When *A* and *B* are sequences, len(*A*) and len(*B*) are the length of those sequences. We found identical peptides using the pairwise2.align.globalxx() code of Biopython^63^, which is a Python module.

### Structural distance and similarity

We calculated structural distances and similarity between proteins using the TM-score tool^64^. This tool provides the root-mean-square deviation (RMSD) of atomic positions as structural distance and the probability of two proteins having the same fold (TM-score) as a structural similarity. When a protein had multiple structures, we estimated distance and similarity for all possible structure pairs from each protein without an identical structure. Finally, we averaged values in order to determine the structural distance or structural similarity of a protein pair.

### Function and phenotype analyses

To analyze the biological relevance of DMI subfamily members, we prepared protein-centric annotations such as protein-function, protein-human disease, protein-yeast phenotype, and protein-chemical compound. To reduce errors introduced by the enrichment tests, we discarded DMI subfamily-enriched term relationships when fewer than three members were associated with a term.

#### Calculation of kappa scores

We adopted Cohen’s kappa as a functional similarity between genes^65^. To calculate kappa scores for pairs of subfamily members, we first established the integrative function annotations including gene-ontology (GO) - biological process (BP), GO-molecular function (MF), GO-cellular component (CC), and KEGG. We constructed a matrix consisting of the relationship between genes (rows) and terms (columns), and we compared the annotation profiles of genes, which were combinations of 1 s (known) and 0 s (unknown)^24^. Note that a cell of the matrix contains 1 or 0, when a gene is annotated with a term or not, respectively. Higher scores indicate that two proteins are more functionally similar or more relevant. In the annotation profile for gene A and gene B, when *a* and *d* are the numbers of agreements (1/1 and 0/0), and *b* and *c* are the numbers of disagreements (1/0 and 0/1), respectively, then the observed proportionate agreement is:

2 $${p}_{o}=\frac{a+d}{a+b+c+d}$$

The expected probability that would be known at random is:

3 $${p}_{known}=\frac{a+b}{a+b+c+d}\cdot \frac{a+c}{a+b+c+d}$$

Similarly:

4 $${p}_{unknown}=\frac{c+d}{a+b+c+d}\cdot \frac{b+d}{a+b+c+d}$$

The overall random agreement probability is:

5 $${p}_{e}={p}_{known}+{p}_{unknown}$$

The kappa score is:

6 $${\rm{\kappa }}=\frac{{p}_{o}-{p}_{e}}{1-{p}_{e}}$$

#### Statistical enrichment test

We adopted a hypergeometric test to implement enrichment tests by using the stats.hypergeom.sf() function in the Python SciPy package (www.scipy.org) to calculate enrichment *p*-values for all enrichment tests. The *p*-values were corrected with Bonferroni correction^66^.

### Chemical compound analysis

#### Construction of DMI subfamily-chemical compound networks

When a DMI subfamily member was associated with at least one compound, we performed an enrichment test with the hypergeometric test to construct the unfiltered DCN (corrected *P* < 0.01). Then we filtered out DC interactions generated by only one DMI subfamily member.

#### Collecting 3D structures including DMIs and chemicals

We curated 3D structures of DMIs from a database of three-dimensional interacting domains (3DID)^67^. Then we collected 165 non-redundant structures with DMI-chemical associations that were composed of 61 DMIs and 85 chemicals. LigandExpo (http://ligand-expo.rcsb.org/index.html) in RCSB was used to map the chemical identifiers in PDB files into SMILE ID (DB and chemical component identifier correspondences: cc-to-pdb.tdd, SMILES and chemical component identifier mappings: *.smi files). PyMOL (http://pymol.org) was used for visualizing the interface of protein 3D structures and chemicals.

#### Calculating minimum distances between interfacial residues of DMIs and chemicals

We selected the minimum value from the calculated distances of each interfacial residue with chemicals. To calculate the distance between a residue and a chemical, distances between the Cα atom of the amino acid and all atoms of the chemical were calculated and the minimum was chosen. The interfacial residues of DMIs were retrieved from the 3did_interface_flat.gz file of the 3DID database. This file contained the residue accessibilities as calculated by NACCESS^68^. We performed permutation tests to investigate the significance of structural distances. We randomly assigned interfacial residues and calculated structural distances 10,000 times to construct a random distribution of such distances for each domain interaction interface and chemical compound relationship. Next, we counted the number of cases (n) in which random values were shorter than the observed values among 10,000 random values. Then we calculated the *p*-value for the significance of the observed distance as *p* = n/10,000. Finally, we converted the *p*-value to a *z*-score. Because 10 Å was already adopted as a threshold in other research^69,70^, we selected, from the 165 structures, the 88 relationships which had a distance < 10 Å and *z* < −1.65. These 88 relationships were used as our reference-DC interactions.

### Statistics

#### Comparison of distributions

To compare the non-normal distributions of the values (e.g., kappa score) between DMI subfamilies and random sets, we adopted a Wilcoxon signed-rank test, because a DMI can have an observed value and a random value. The *p*-value of this test indicates the significance of the difference between the rank distributions from DMI and rDMI.

## Supplementary information

Supplementary Information.

Supplementary table S1.

Supplementary table S2.

Supplementary table S3.

Supplementary table S4.

Supplementary table S5.

Supplementary table S6.

Supplementary table S7.

Supplementary table S8.

## Data Availability

All data generated or analyzed during this study are provided in this published article and *Supplementary Information* Files.
