# Supplementary material for: Domain-mediated interactions for protein subfamily identification
Source: Sci Rep. 2020 Jan 14;10:264. doi: 10.1038/s41598-019-57187-z (PMC6959277; doi:10.1038/s41598-019-57187-z)
Supplement: Supplementary file 1 — Supplementary Information. [file 41598_2019_57187_MOESM1_ESM.pdf]

Domain-mediated interactions for protein subfamily identification

Heetak Lee[1], Inhae Kim[1], Seong Kyu Han[1], Donghyo Kim[1], Jungho Kong[1], and Sanguk Kim[1]*

[1]Department of Life Sciences, Pohang University of Science and Technology, Pohang 790-784, Korea

Supplementary Figures

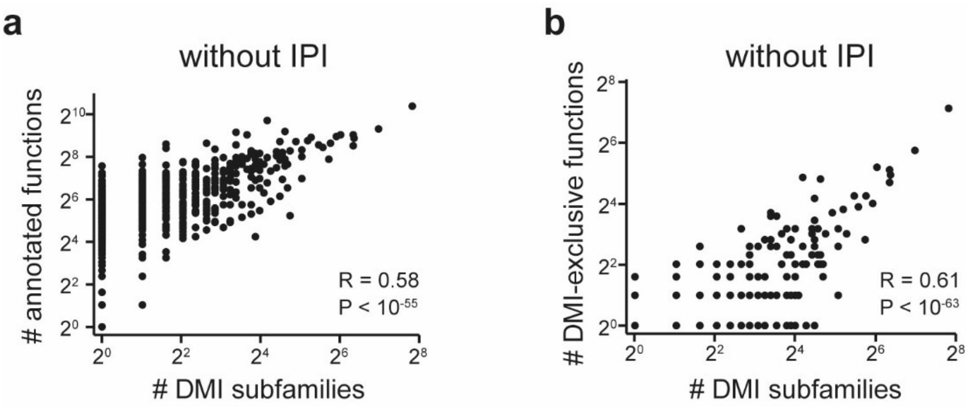

Supplementary Figure S1. Correlations between the numbers of DMI subfamilies and functional divergence based on functional annotations excluding annotations bearing the "Inferred from Physical Interaction" (IPI) evidence code. (a) Correlation between the number of unique annotated functions of parent-domain family members and the number of DMI subfamilies contained in the parent-domain family. (b) Correlation between the number of DMI-exclusive functions and the number of DMI subfamilies within the parent-domain families.

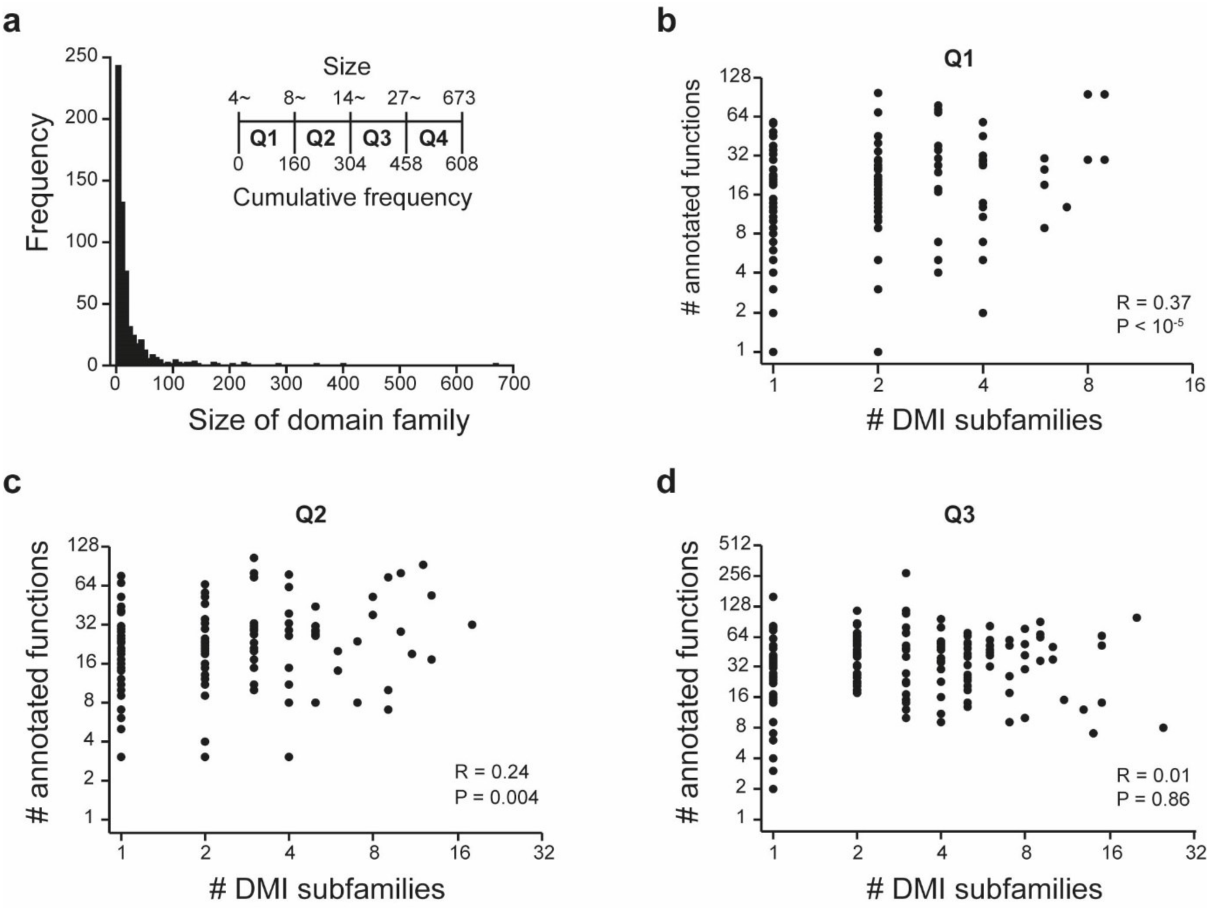

Supplementary Figure S2. Correlations between the numbers of DMI subfamilies and annotated functions for individual parent-domain families. (**a**) Size distribution of parent-domain families. Parent-domain families were divided into four groups according to size. (**b**-**d**) Correlations between the number of DMI subfamilies and annotated functions (Spearman correlation) (**b**) in Q1; (**c**) in Q2; (**d**) in Q3.

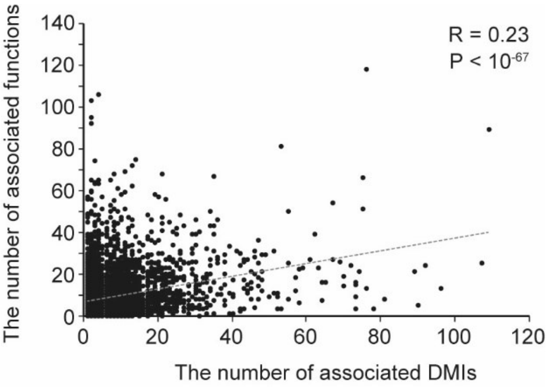

Supplementary Figure S3. Correlations between the numbers of associated DMIs and annotated functions for individual proteins (Spearman correlation).

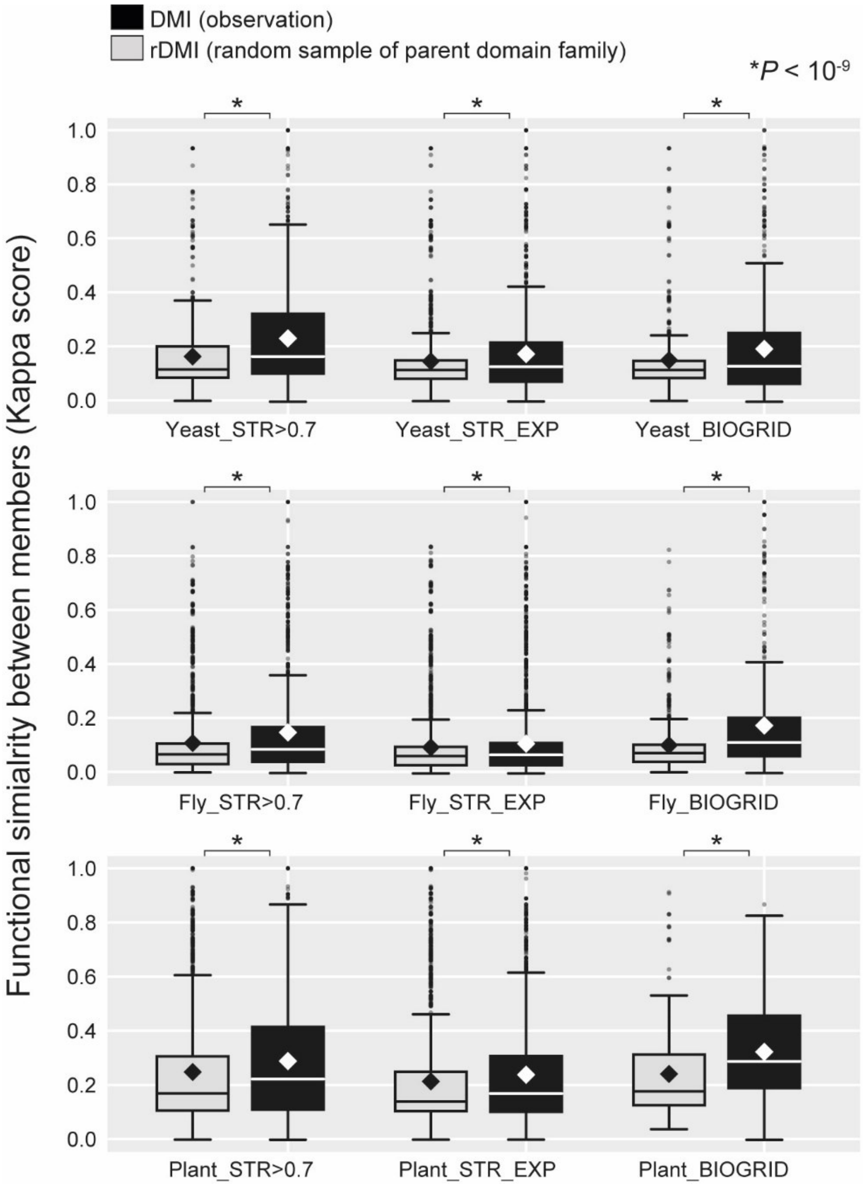

Supplementary Figure S4. Functional similarity between members within DMI subfamilies from yeast, fly, and plant interactomes. Comparisons between DMI subfamily and random samples of parent-domain family. Gray and black boxplots indicate distributions of values from random samples and DMI subfamilies, respectively. Diamonds show mean; *p*-values were calculated by Wilcoxon signed-rank test.

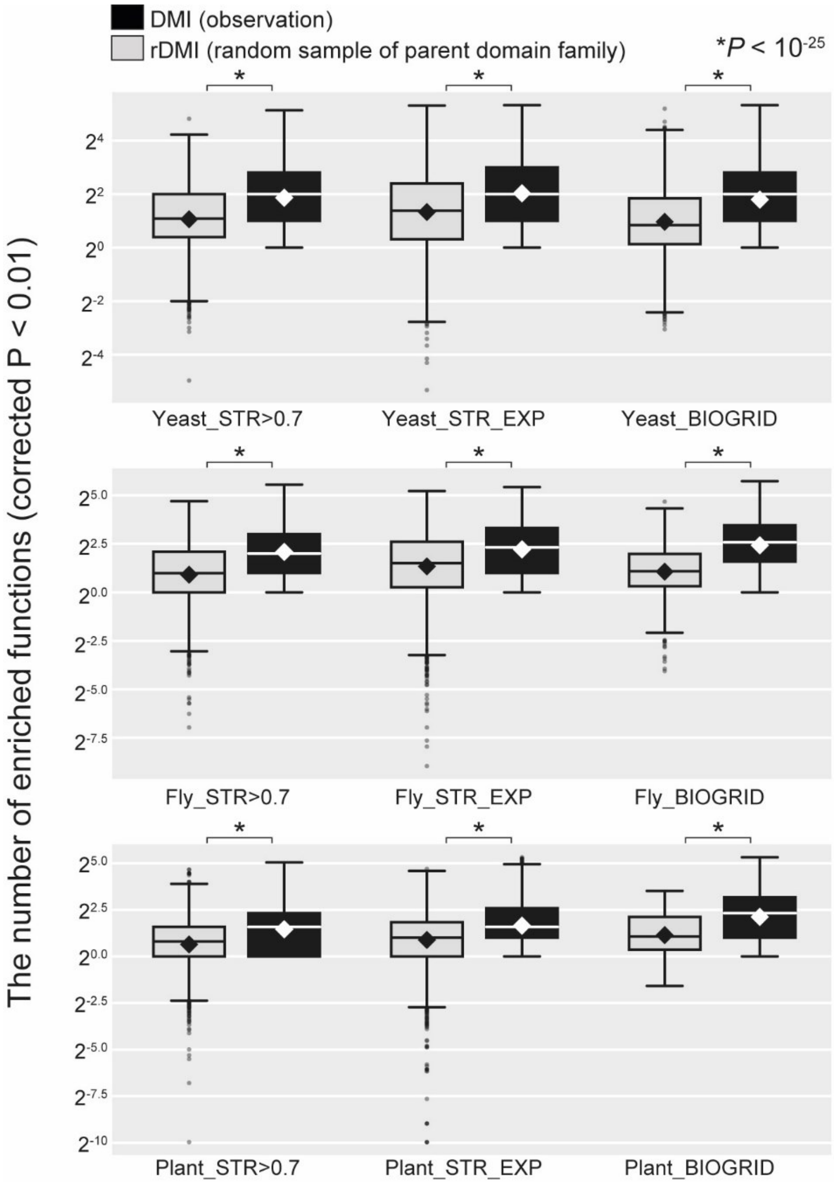

Supplementary Figure S5. The number of enriched terms for DMI subfamilies from yeast, fly, and plant interactomes. Comparisons between DMI subfamilies and random samples of parent-domain family. Gray and black boxplots indicate distributions of values from random samples and DMI subfamily, respectively. Diamonds show mean; *p*-values were calculated by Wilcoxon signed-rank test.

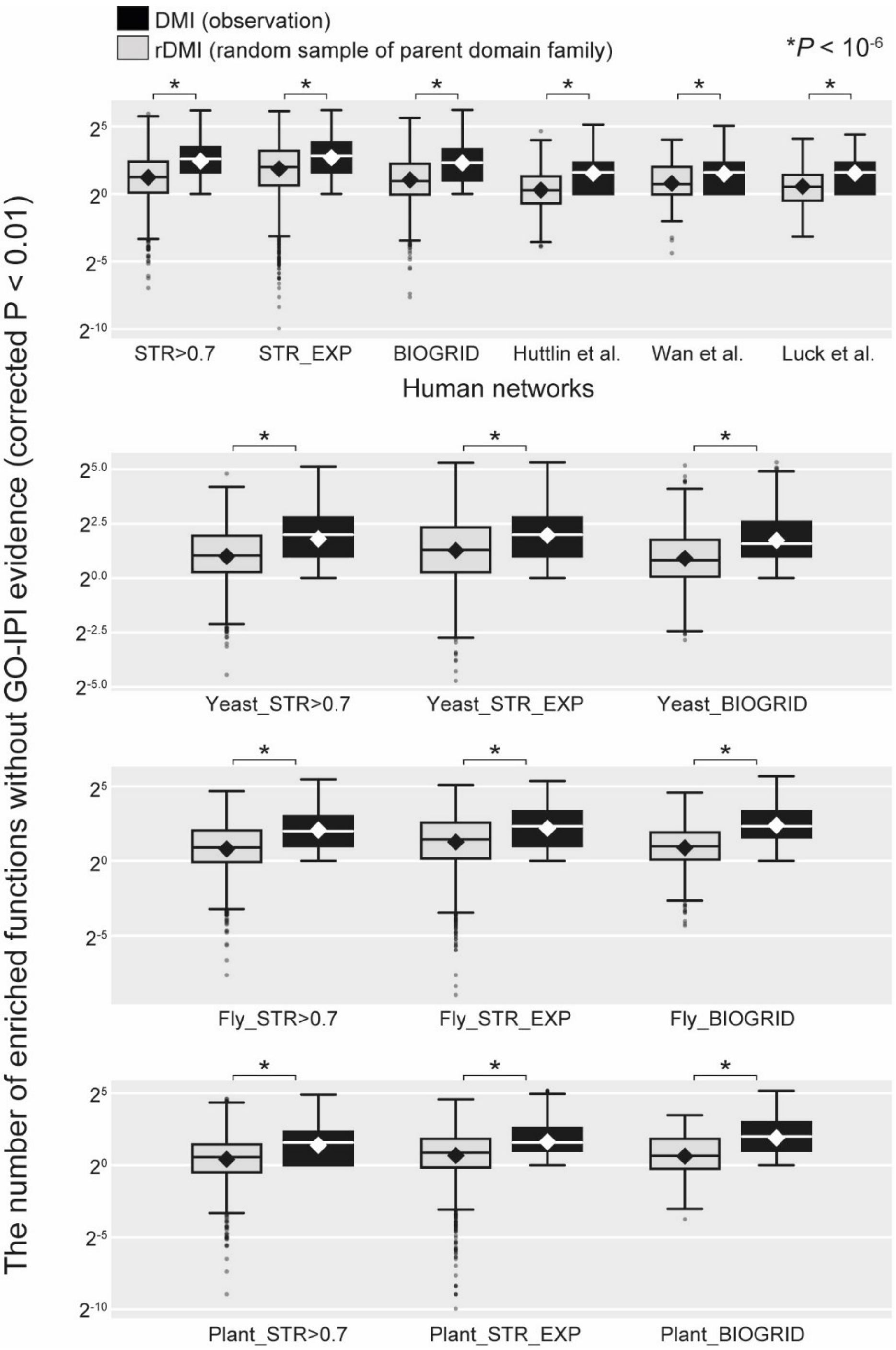

Supplementary Figure S6. The number of enriched terms using integrative functional annotations excluding the "IPI" code of gene ontology for DMI subfamilies from human, yeast, fly, and plant interactomes. Comparisons between DMI subfamilies and random samples of parent-domain family. Gray and black boxplots indicate distributions of values from random samples and DMI subfamily, respectively. Diamonds show mean; and *p*-values were calculated by Wilcoxon signed-rank test.

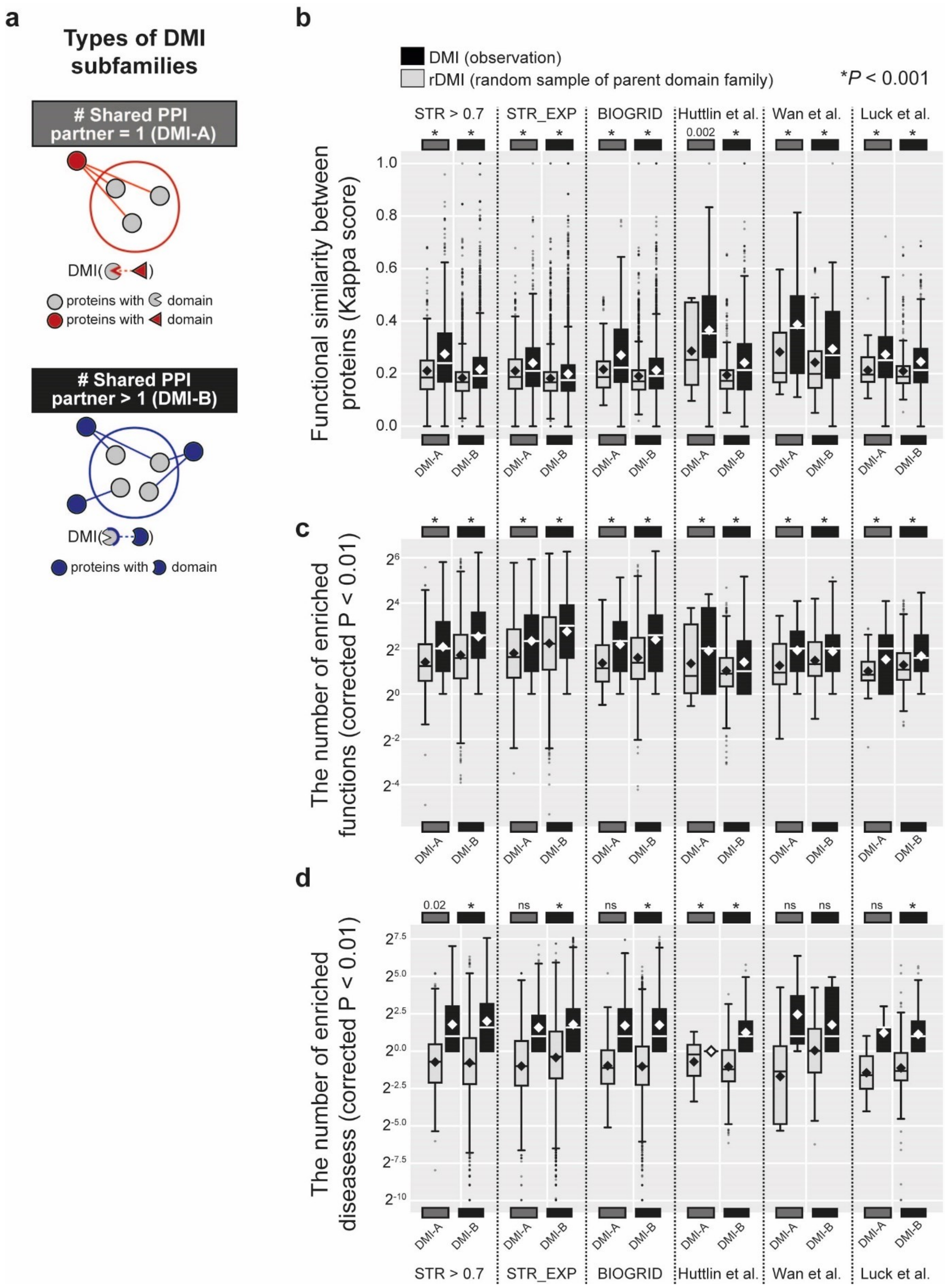

Supplementary Figure S7. DMI subfamilies as biological subcategories of parent-domain family.
(**a**) Scheme for types of DMI subfamilies. DMI-A contains DMI subfamilies consisting of proteins which

share only one protein interaction partner. DMI-B contains DMI subfamilies consisting of proteins which share more than one protein interaction partner. Shared protein interaction partners must contribute to the corresponding DMI. (**b**-**d**) Comparisons between DMI subfamily (DMI) and random samples of parent-domain family (rDMI). Gray and black boxplots indicate distributions of values from random samples and DMI subfamily, respectively. Diamonds show mean; *p*-values were calculated by Wilcoxon signed-rank test. Several networks were used to detect DMI subfamilies: STR>0.7 (a STRING network containing links with a combined score greater than 0.7); STR_EXP (a STRING network containing links with an experimental score greater than zero); BIOGRID (involving experimentally-confirmed links); and Huttlin et al., Wan et al., and Luck et al. (networks constructed through AP-MS, co-fractionation, and yeast two-hybrid, respectively) (*$P < 0.001$, “ns” is non-significant). (**b**) Functional similarity between members within each subfamily. (**c**) The number of enriched functional terms. (**d**) The number of enriched diseases.

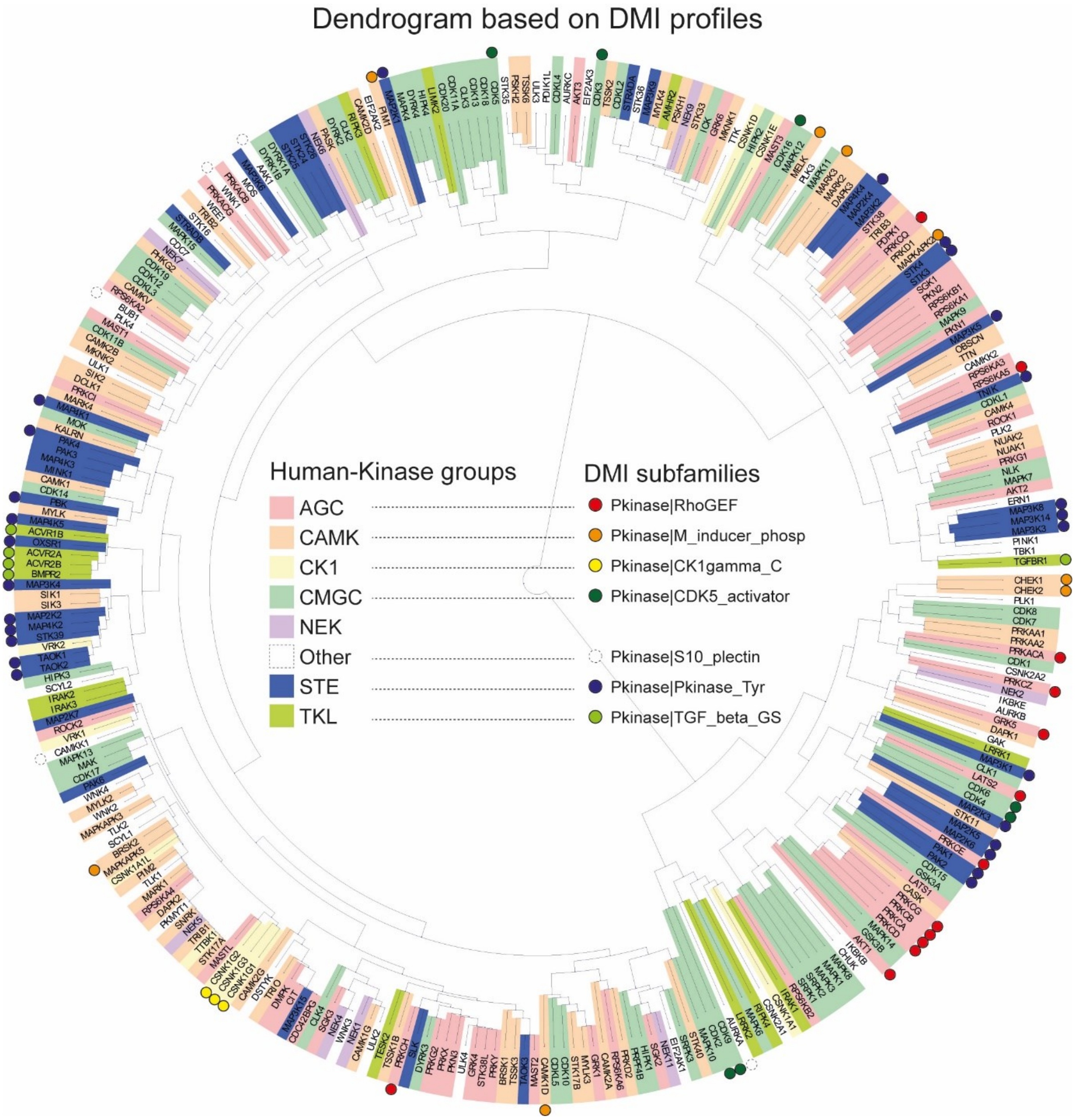

Supplementary Figure S8. Dendrogram based on DMI profile similarity between proteins. Each protein has a vector consisting of 0 (not associated with a DMI) or 1 (associated with a DMI) and the distance between proteins. To cluster proteins, the Ward variance minimization algorithm was implemented. Pink, apricot, beige, mint, lavender, white, blue, and lime colors indicate involved kinase groups. Colored circles show proteins; proteins overlapping between kinase group and the DMI subfamily are connected with dotted lines (corrected $P < 0.05$ in Fig. 3).

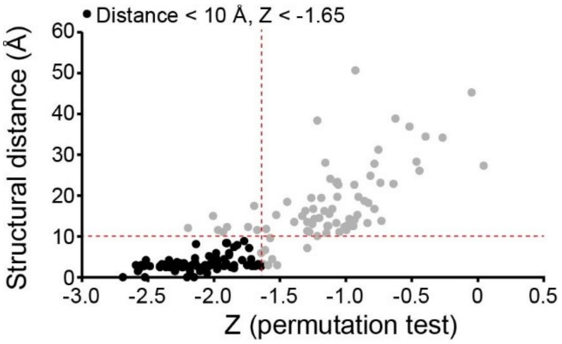

Supplementary Figure S9. Structural proximity between domain interaction interface and chemical compound. Among 165 non-redundant structures, 88 DC interactions were selected having a structural distance under 10 Å with *Z* < -1.65. Black dots indicate the reference-DC interactions.

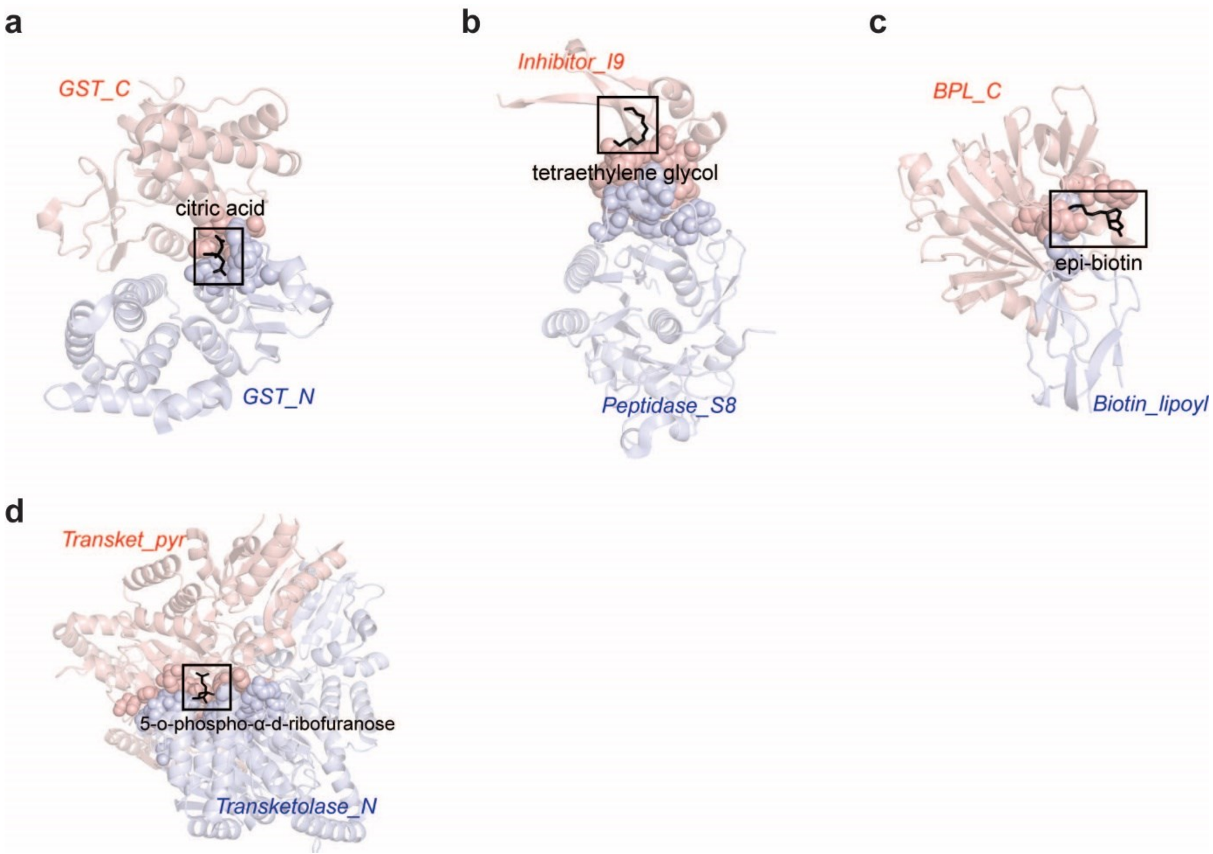

Supplementary Figure S10. Examples of DMI interface and ligand-binding pocket. Complex structures of (**a**) GST_C/GST_N-citric acid interaction (PDB: 1YY7, structural distance (*D*) = 3.70 Å). (**b**) Inhibitor_I9/Peptidase_S8-tetraethylene glycol interaction (PDB: 4OV6, *D* = 4.50 Å). (**c**) BPL/Biotin_lipoyl-epi-biotin interaction (PDB: 2EJF, *D* = 3.73 Å). (**d**) Transket_pyr/Trans-ketolase_N-5-o-phospho-alpha-d-ribofuranose interaction (PDB: 2R5N, *D* = 5.05 Å).

Legends for Supplementary Tables

Supplementary Table S1. Network statistics. Numbers of nodes, links, and associated DMI types from the given networks (total: 15 networks).

Supplementary Table S2. Subfamily statistics. Numbers of parent-domain families and DMI subfamilies in the given networks.

Supplementary Table S3. DMI-exclusive functions. The relationship between DMI subfamily and functional terms within GO BP excluding IPI evidence (hypergeometric test, corrected $P < 0.01$). Each column contains DMI subfamily, enriched functions, corrected *p*-value, the number of overlapping genes, and list of genes overlapping between DMI subfamily and functional module.

Supplementary Table S4. Values of boxplots. Because various networks, species, DMI classifications, and biological categories were considered, this table summarizes 160 cases with means, *p*-values, and mean differences (observed value – expected value).

Supplementary Table S5. Disease enrichment results. Relationships between DMI subfamilies and diseases (hypergeometric test, corrected $P < 0.01$). The relationships mentioned in the text are marked with a “Y” in the “Mentioned” column of the table.

Supplementary Table S6. DMI-A and DMI-B statistics. DMI subfamilies were classified into DMI-A (with a single PPI partner) and DMI-B (with multiple PPI partners). This table summarizes the numbers of DMI-A and DMI-B subfamilies from 15 independent sets of DMI subfamilies.

Supplementary Table S7. DMI-chemical compound network. Results of enrichment tests between DMI subfamilies and chemical compounds (hypergeometric test, $P < 0.01$).

Supplementary Table S8. DC interactions with structural evidence. There are approximately 300 DMI subfamily-chemical compound interactions with PDB IDs. The “-log (P)” and “Structural distance” entries in this table are associated with Figure 4d.
